# Fulvestrant with or without anti‐HER2 therapy in patients in a postmenopausal hormonal state and with ER‐positive HER2‐positive advanced or metastatic breast cancer: A subgroup analysis of data from the Safari study (JBCRG‐C06)

**DOI:** 10.1002/cam4.6390

**Published:** 2023-08-01

**Authors:** Misato Masuyama, Norikazu Masuda, Hidetoshi Kawaguchi, Yutaka Yamamoto, Shigehira Saji, Takahiro Nakayama, Kenjiro Aogi, Keisei Anan, Shoichiro Ohtani, Nobuaki Sato, Toshimi Takano, Eriko Tokunaga, Seigo Nakamura, Yoshie Hasegawa, Masaya Hattori, Tomomi Fujisawa, Satoshi Morita, Miki Yamaguchi, Toshinari Yamashita, Daisuke Yotsumoto, Masakazu Toi, Shinji Ohno

**Affiliations:** ^1^ Department of Breast and Endocrine Surgery, Graduate School of Medicine Osaka University Osaka Japan; ^2^ Department of Surgery, Breast Oncology National Hospital Organization Osaka National Hospital Osaka Japan; ^3^ Department of Breast and Endocrine Surgery Nagoya University Graduate School of Medicine Nagoya Japan; ^4^ Department of Breast Surgery Matsuyama Red Cross Hospital Matsuyama Japan; ^5^ Department of Breast and Endocrine Surgery Kumamoto University Hospital Kumamoto Japan; ^6^ Department of Medical Oncology Fukushima Medical University Fukushima Japan; ^7^ Department of Breast and Endocrine Surgery Osaka International Cancer Institute Osaka Japan; ^8^ Department of Breast Oncology NHO Shikoku Cancer Center Matsuyama Japan; ^9^ Department of Surgery Kitakyushu Municipal Medical Center Kitakyushu Japan; ^10^ Department of Breast Surgery Hiroshima City Hiroshima Citizens Hospital Hiroshima Japan; ^11^ Department of Breast Oncology Niigata Cancer Center Hospital Niigata Japan; ^12^ Department of Medical Oncology Toranomon Hospital Tokyo Japan; ^13^ Department of Breast Oncology NHO Kyushu Cancer Center Fukuoka Japan; ^14^ Department of Surgery, Division of Breast Surgical Oncology Showa University School of Medicine Tokyo Japan; ^15^ Department of Breast Surgery Hachinohe City Hospital, Hachinohe Japan; ^16^ Department of Breast Oncology Aichi Cancer Center Hospital Nagoya Japan; ^17^ Department of Breast Oncology Gunma Prefectural Cancer Center Ohta Japan; ^18^ Department of Biomedical Statistics and Bioinformatics Kyoto University Graduate School of Medicine Kyoto Japan; ^19^ Department of Breast Surgery JCHO Kurume General Hospital Kurume Japan; ^20^ Department of Breast Surgery and Oncology Kanagawa Cancer Center Yokohama Japan; ^21^ Department of Breast and Thyroid Surgery Hakuaikai Social Medical Corporation, Sagara Hospital Kagoshima Japan; ^22^ Department of Breast Surgery Kyoto University Graduate School of Medicine Kyoto Japan; ^23^ Breast Oncology Center The Cancer Institute Hospital of JFCR Tokyo Japan

**Keywords:** fulvestrant, HER2‐positive advanced or metastatic breast cancer, real‐world evidence, time to chemotherapy

## Abstract

**Background:**

The role of endocrine therapy in the treatment of patients in a postmenopausal hormonal state and with estrogen receptor (ER)‐positive, human epidermal growth factor receptor 2 (HER2)‐positive advanced or metastatic breast cancer (AMBC) is unclear.

**Methods:**

We analyzed the data from 94 patients with ER‐positive HER2‐positive AMBC enrolled in the Safari study (UMIN000015168), a retrospective cohort study of 1072 ER‐positive AMBC patients in a postmenopausal hormonal state who received fulvestrant 500 mg (F500): (1) to compare time to treatment failure (TTF) and overall survival (OS) by treatment group, and TTF by treatment line; (2) in patients who received endocrine therapy (including F500) or anti‐HER2 therapy as initial systemic therapy before chemotherapy, to investigate relations between TTF for the first‐line therapy or time to chemotherapy (TTC) and OS; (3) to investigate factors associated with OS.

**Results:**

The TTF was longer in the patients treated with F500 as first‐ or second‐line therapy (*n* = 20) than in those who received later‐line F500 therapy (*n* = 74) (6.6 vs. 3.7 months; HR, 1.98; *p* = 0.014). In the 59 patients who received endocrine therapy or anti‐HER2 therapy as initial systemic therapy before chemotherapy, those with TTC ≥3 years had longer median OS than those with TTC <3 years (10.5 vs. 5.9 years; HR, 0.32; *p* = 0.001). Longer TTC was associated with prolonged OS.

**Conclusions:**

In patients with ER‐positive HER2‐positive AMBC enrolled in the Safari study, TTF was longer in patients who received F500 as first‐ or second‐line therapy. In patients who received chemotherapy‐free initial systemic therapy, the prolonged OS in those with TTC ≥3 years suggests that this value may be a helpful cut‐off for indicating clinical outcomes.

## Graphical abstract

A subgroup analysis of the Safari study was carried out to investigate clinical outcomes in patients in a postmenopausal hormonal state and with estrogen receptor‐positive, human epidermal growth factor receptor 2 (HER2)‐positive advanced or metastatic breast cancer who received fulvestrant 500 mg (F500) with or without anti‐HER2 therapy (*n* = 94). Time to treatment failure (TTF) was longer in patients who received F500 as first‐ or second‐line therapy, suggesting that those for whom advanced treatment lines are needed have disease resistance to both endocrine and anti‐HER2 therapies. In the 59 patients who received endocrine therapy (including F500) or anti‐HER2 therapy as initial systemic therapy before being switched to chemotherapy, median overall survival was longer in those with time to chemotherapy (TTC) ≥3 years, suggesting that this may be a helpful cut‐off value for indicating clinical outcomes.

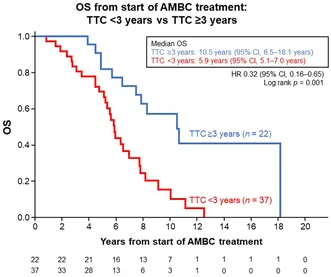

## 1 INTRODUCTION

Fulvestrant is a selective estrogen receptor (ER) degrader indicated for the treatment of postmenopausal patients with ER‐positive advanced breast cancer and disease progression after antiestrogen therapy. Fulvestrant 500 mg (F500) has been shown to be more efficacious than the aromatase inhibitor (AI) anastrozole 1 mg as first‐line endocrine therapy for postmenopausal women with ER‐positive advanced or metastatic breast cancer (AMBC).^1^, ^2^

The Safari study (JBCRG‐C06, UMIN000015168) was a large‐scale multicenter retrospective cohort study carried out to investigate clinical outcomes in patients in a postmenopausal state and with ER‐positive AMBC who received treatment with F500 in Japan. The results have been reported previously.^3^ Briefly, the study population comprised 1072 patients, and the median time to treatment failure (TTF) was 5.4 months. The results of multivariate analysis showed that earlier F500 use, a longer period from AMBC diagnosis to F500 use, and no prior palliative chemotherapy were associated with significantly longer TTF. The latter two factors were also found to be associated with prolonged overall survival (OS),^4^ and in the subgroup of human epidermal growth factor receptor 2 (HER2)‐negative patients receiving F‐500 as second‐ or later‐line therapy, with prolonged TTF.^5^

Few clinical trials have focused on the subgroup of postmenopausal patients with ER‐positive HER2‐positive AMBC; therefore, limited data are available on the efficacy of endocrine therapy in this population. Owing to the higher biological grade of HER2‐positive breast cancer and with the treatment goal of OS prolongation, the standard therapy for ER‐positive HER2‐positive AMBC is widely recognized as the combination of chemotherapy and anti‐HER2 therapy.^6^, ^7^ By contrast, in cases of small tumor volume and low proliferative activity (i.e., slow growth), endocrine therapy prior to chemotherapy is chosen for some patients, to maintain their quality of life or based on individual patient preference (to avoid chemotherapy‐related adverse events). However, the usefulness of endocrine therapy with or without anti‐HER2 therapy as initial systemic therapy before switching to chemotherapy remains uncertain. To address this research gap, we carried out a subgroup analysis using data for ER‐positive HER2‐positive AMBC patients enrolled in the Safari study. We investigated TTF and OS in all these patients, and TTF, OS, and time to chemotherapy (TTC) in those for whom endocrine therapy (including F500) or anti‐HER2 therapy was chosen as initial systemic therapy before chemotherapy. Additionally, we investigated factors associated with outcomes.

## 2 PATIENTS AND METHODS

### 2.1 Study design

The Safari study (UMIN000015168) was a retrospective multicenter cohort study using data from >1000 ER‐positive AMBC patients in a postmenopausal hormonal state (through natural changes, or in the case of premenopausal patients, under concomitant treatment with a luteinizing hormone‐releasing hormone analog), who received treatment with F500 at 16 sites in Japan between November 25, 2011 (the date on which F500 was approved in Japan), and December 31, 2014. Patients who received F500 plus anti‐HER2 therapy (i.e., trastuzumab), denosumab, and zoledronic acid were included in the Safari study, whereas patients were excluded if they received F500 in combination with other endocrine therapies and/or chemotherapies and/or nontrastuzumab targeted therapies. Further details of the study design, and a description of the patient cohort, are available in a previous publication.^3^

### 2.2 Definition of AMBC, hormone receptor status, and HER2 status

AMBC was defined as locally advanced unresectable, de novo metastatic, or recurrent metastatic breast cancer (i.e., found after an initial diagnosis of nonmetastatic disease). Tumors were considered ER‐positive if ≥10% (before the 2010 introduction of the ASCO/CAP Guideline Recommendations^8^) or ≥1% (after 2010, in accordance with the ASCO/CAP Guideline Recommendations^8^) of the cells showed positive immunohistological staining, or if ER positivity was shown by a biological method such as ligand binding assay. HER2 overexpression was determined by immunohistological staining and in situ hybridization (ISH), in accordance with the 2013 ASCO/CAP Guideline Recommendations.^9^

A membrane‐staining score of 3+ was recorded as an HER2‐positive result and a score of 1+ or 0 as HER2‐negative. Fluorescence in situ hybridization (FISH) was carried out if the immunohistochemical HER2 score was equivocal (i.e., 2+). A HER2‐positive FISH result was regarded as confirmatory even if the ISH score was 1+.

### 2.3 Follow‐up

Patients were followed up according to the National Comprehensive Cancer Network Guidelines for Invasive Breast Cancer (Version 4.2018),^10^ which is standard care in Japan.

### 2.4 Subgroup analyses

We report the results of subgroup analyses of data from the Safari study. Three analyses were carried out to investigate clinical outcomes in the subgroup of patients with ER‐positive HER2‐positive AMBC who received F500 with or without anti‐HER2 therapy.

Data for TTF and OS were analyzed and the results were compared across subgroups. TTF was defined as the time from the start of F500 treatment to its discontinuation. Details of the definitions of treatment failure are available in the main Safari study report (see Figure S1 of Ref. 3). Briefly, treatment failure included discontinuation of treatment due to confirmed deterioration (e.g., increase in levels of tumor markers, worsening of symptoms, progressive disease, or death), switch to palliative care or another treatment, and change of treatment due to adverse effects or at the patient's request. OS was defined as the time from the start of first‐line AMBC treatment to death. TTF from the start of first‐line AMBC treatment and OS from the start of F500 treatment were also examined, for exploratory purposes.

#### 2.4.1 Analysis 1

Data from all the ER‐positive HER2‐positive AMBC patients (*n* = 94) were analyzed to compare TTF (time from the start of F500 treatment to its discontinuation) and OS by treatment group (F500 alone vs. F500 plus anti‐HER2 therapy), and TTF by treatment line (first‐ or second‐line vs. third‐ or later‐line).

#### 2.4.2 Analysis 2

Data from the ER‐positive HER2‐positive AMBC patients who received endocrine therapy (including F500) or anti‐HER2 therapy as initial systemic therapy for AMBC before being switched to chemotherapy due to disease progression (*n* = 59) were analyzed to investigate relations between TTC and OS. A cut‐off value of 3 years for TTC was used.

#### 2.4.3 Analysis 3

Factors associated with OS were investigated in the ER‐positive HER2‐positive AMBC patients who received endocrine therapy (including F500) or anti‐HER2 therapy as initial systemic therapy for AMBC before being switched to chemotherapy (*n* = 59). Additionally, after excluding data from 8 patients with de novo stage IV cancer, data from the remaining 51 patients were analyzed because the biological characteristics of recurrent cancer differ from those of stage IV disease. For reference, factors associated with OS were investigated in all the ER‐positive HER2‐positive AMBC patients who received F500 with or without anti‐HER2 therapy (*n* = 94).

### 2.5 Statistical analyses

The Kaplan–Meier method was used to estimate TTF, TTC, and OS, and differences between the survival curves were analyzed by the log‐rank test. The Cox proportional hazards model was used to evaluate the relationship between each clinicopathological factor and OS. All tests were two‐sided, and *p* < 0.05 was considered statistically significant.

## 3 RESULTS

### 3.1 Patients

**FIGURE 1:**
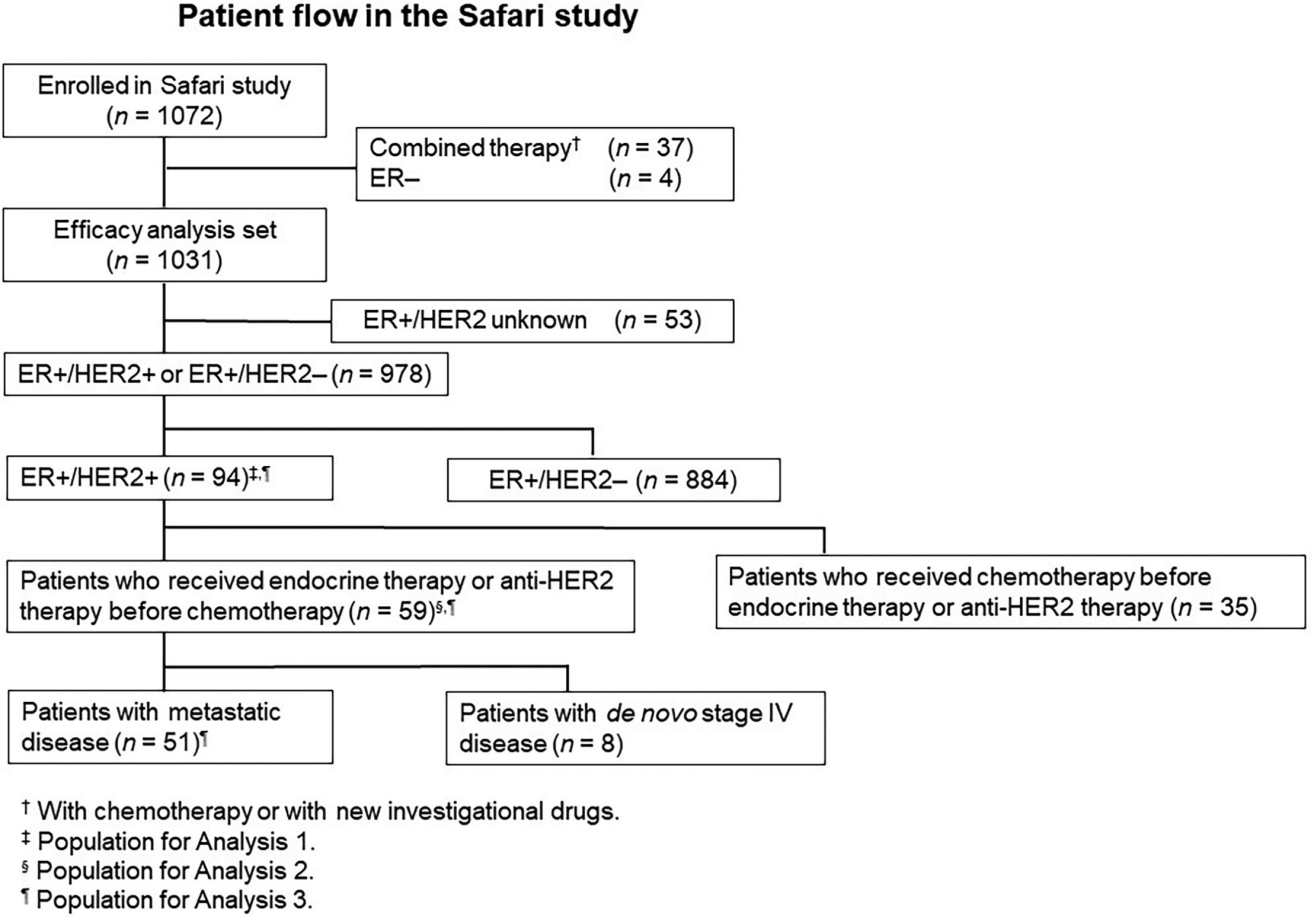
Patient flow in the Safari study. ER, estrogen receptor; HER2, human epidermal growth factor receptor 2.

Of the 1072 patients enrolled in the Safari study, 94 were confirmed as having ER‐positive HER2‐positive AMBC, and their data were included in the present subgroup analysis. Of them, 59 patients received endocrine therapy (including F500) or anti‐HER2 therapy as initial systemic therapy for AMBC before being switched to chemotherapy (Figure 1).

The baseline characteristics of the 94 patients are summarized in Table 1. Their median age was 57.5 years at diagnosis of AMBC and 61 years at the start of F500 treatment, and 35 (37%) had visceral metastases. Most of the patients with ER‐positive HER2‐positive AMBC (74/94, 79%) received F500 as third‐ or later‐line therapy; only a small minority (2/94, 2%) received it as first‐line therapy. A total of 52 were treated with F500 alone, and 42 with the combination of F500 plus anti‐HER2 therapy (i.e., trastuzumab). There were no significant differences between these two subgroups in terms of patient characteristics.

**TABLE 1:** Summary of patient characteristics.^a^

	All (*n* = 94)	F500 monotherapy (*n* = 52)	F500 plus trastuzumab (anti‐HER2) (*n* = 42)
Median age at AMBC diagnosis, years (range)	57.5 (33–85)	59 (33–85)	56.5 (39–75)
Median age at start of fulvestrant treatment, years (range)	61 (35–96)	61 (35–96)	61.5 (44–77)
Metastasis diagnosis			
Recurrence after initial curative treatment	74 (79)	38 (73)	36 (86)
De novo stage IV disease	20 (21)	14 (27)	6 (14)
Visceral metastasis at diagnosis			
No	59 (63)	35 (67)	24 (57)
Yes	35 (37)	17 (33)	18 (43)
Hormonal receptor status			
ER(+) PgR(−)	20 (21)	11 (21)	9 (21)
ER(+) PgR(+)	74 (79)	41 (79)	33 (79)
HER2 status			
3+	42 (45)	16 (31)	26 (62)
2+ or 1+ (positive by FISH)	36 (38)	24 (46)	12 (29)
Positive (score unknown)	16 (17)	12 (23)	4 (10)
Fulvestrant treatment line			
1st line	2 (2)	0	2 (5)
2nd line	18 (19)	11 (21)	7 (17)
≥3rd line	74 (79)	41 (79)	33 (79)
Prior chemotherapy before fulvestrant use			
No	41 (44)	26 (50)	15 (36)
Yes	53 (56)	26 (50)	27 (64)
Anthracycline‐based^b^	21 (22)	10 (19)	11 (26)
Taxane‐based^b^	31 (33)	13 (25)	18 (43)
Other	15 (16)	9 (17)	6 (14)
Prior anti‐HER2 therapy before fulvestrant use			
No	41 (44)	34 (65)	7 (17)
Yes	53 (56)	18 (35)	35 (83)
Trastuzumab	53 (56)	18 (35)	35 (83)
Lapatinib^c^	21 (22)	4 (8)	17 (40)
T‐DM1^c^	5 (5)	1 (2)	4 (10)

Abbreviations: AMBC, advanced/metastatic breast cancer; ER, estrogen receptor; FISH, fluorescence in situ hybridization; HER2, human epidermal growth factor receptor 2; PgR, progesterone receptor; T‐DM1, trastuzumab emtansine.

^a^ Values presented as *n* (%), unless otherwise indicated.

^b^ Including duplicated cases.

^c^ Patients received prior treatment with trastuzumab.

### 3.2 Outcomes

#### 3.2.1 Analysis 1: TTF and OS in all ER‐positive HER2‐positive AMBC patients

**FIGURE 2:**
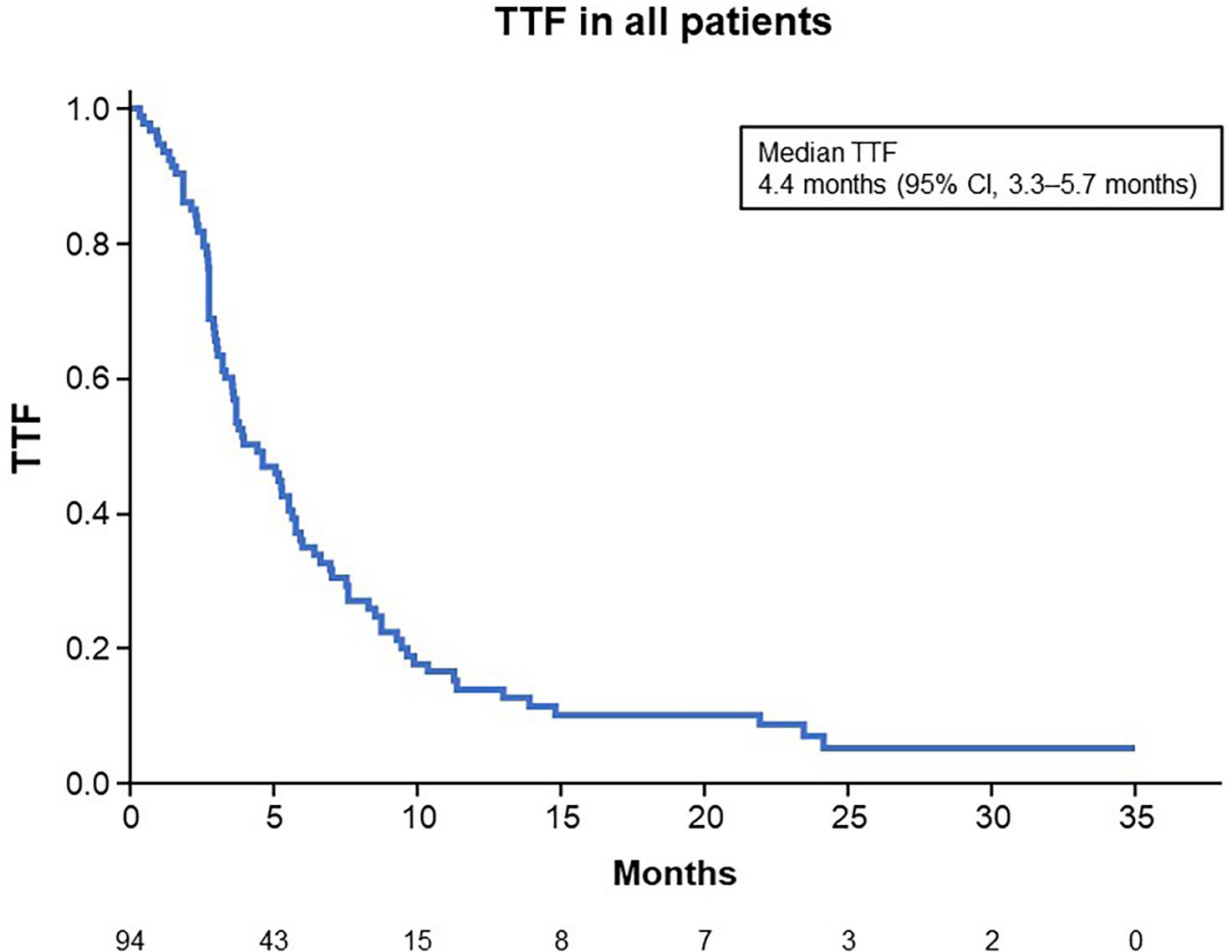
Kaplan–Meier estimates for time to treatment failure (TTF) in all patients (*n* = 94). CI, confidence interval.

**FIGURE 3:**
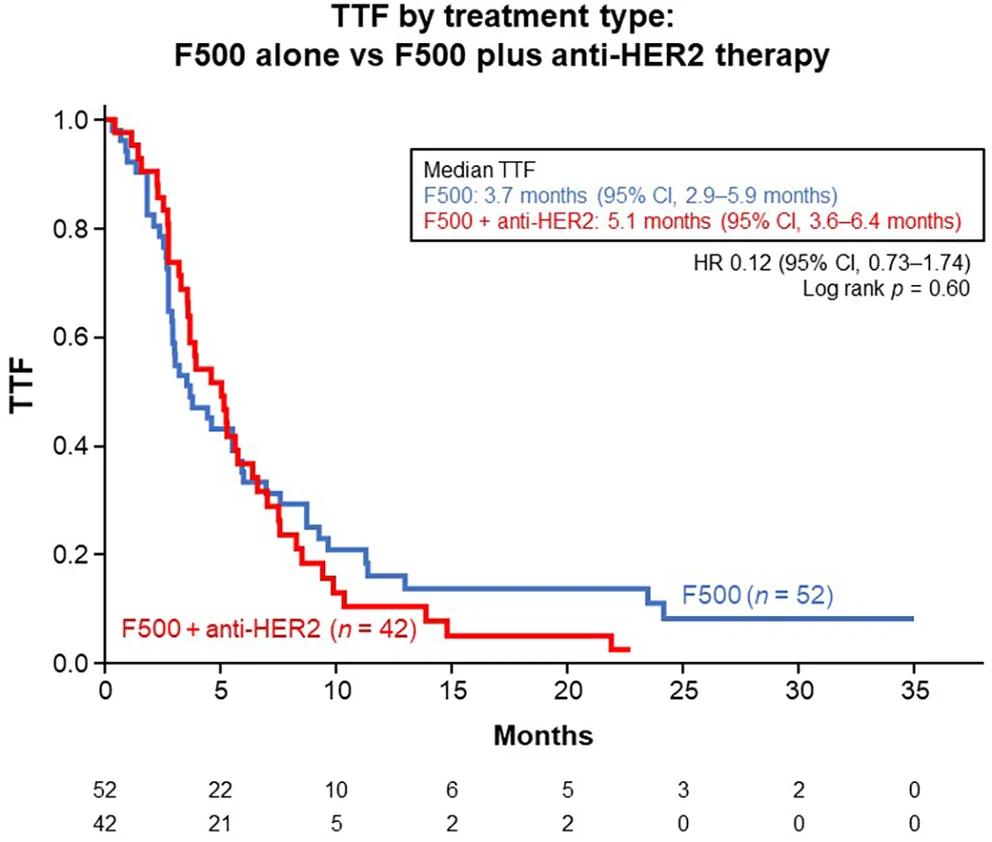
Kaplan–Meier estimates for time to treatment failure (TTF) in all patients (*n* = 94), by treatment type. CI, confidence interval; HR, hazard ratio.

At a median follow‐up of 3.9 years (from the start of treatment with F500), median TTF in all the ER‐positive HER2‐positive AMBC patients who received F500 with or without anti‐HER2 therapy was 4.4 months (95% confidence interval [CI], 3.3–5.7 months) (Figure 2). There was no significant difference in median TTF between patients treated with F500 alone (*n* = 52, 3.7 months [95% CI, 2.9–5.9 months]) and those treated with F500 plus anti‐HER2 therapy (*n* = 42, 5.1 months [95% CI, 3.6–6.4 months]) (Figure 3).

**FIGURE 4:**
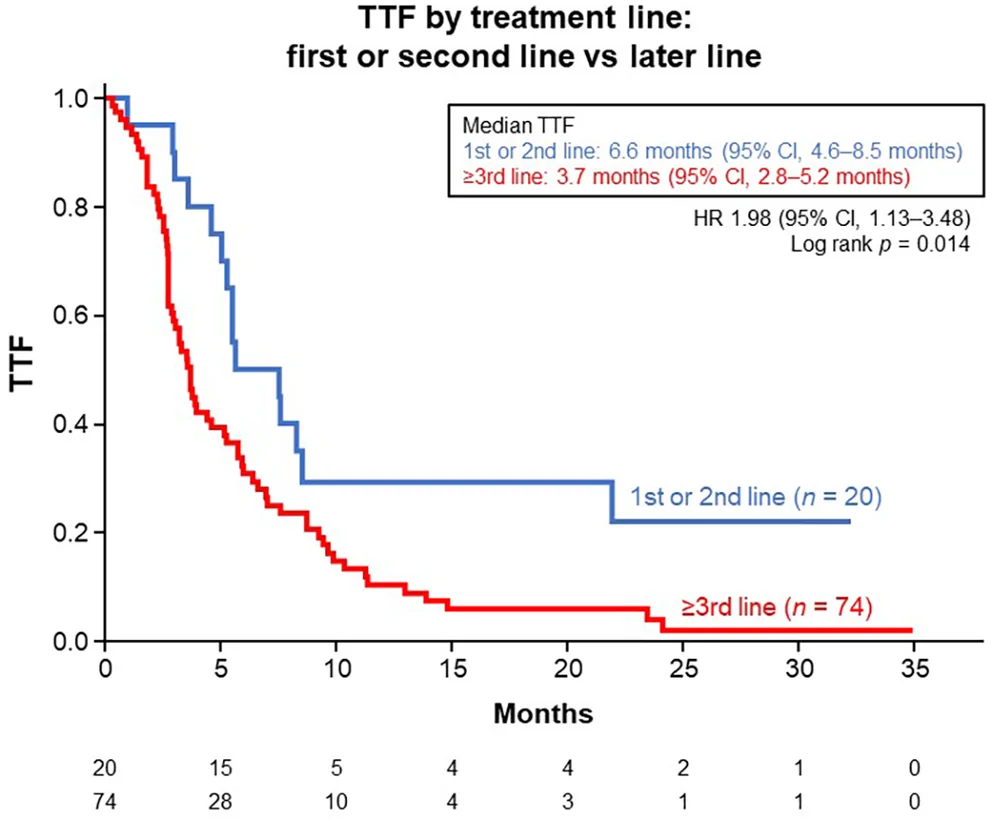
Kaplan–Meier estimates for time to treatment failure (TTF) in all patients (*n* = 94), by treatment line. CI, confidence interval; HR, hazard ratio.

Regarding treatment line, TTF was significantly longer in patients treated with F500 as first‐ or second‐line therapy (*n* = 20) than in those treated with F500 as third‐ or later‐line therapy (*n* = 74), the results being 6.6 months (95% CI, 4.6–8.5 months) and 3.7 months (95% CI, 2.8–5.2 months), respectively (hazard ratio [HR], 1.98 [95% CI, 1.13–3.48]; *p* = 0.014) (Figure 4).

**FIGURE 5:**
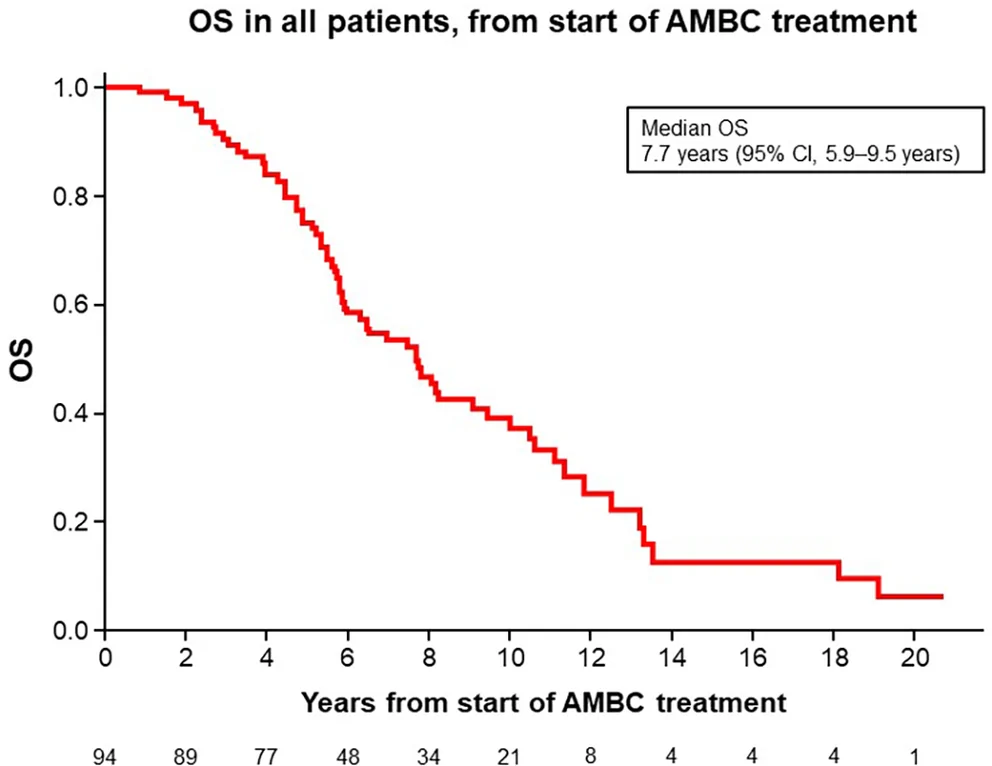
Kaplan–Meier estimates for overall survival (OS) in all patients (*n* = 94), from the start of advanced or metastatic breast cancer (AMBC) treatment. CI, confidence interval.

Median OS was 7.7 years (95% CI, 5.9–9.5 years) from the start of AMBC treatment (Figure 5), and 2.6 years (95% CI, 2.3–3.3 years) from the start of F500 treatment (Figure S1).

#### 3.2.2 Analysis 2: TTC in patients who received endocrine therapy or anti‐HER2 therapy as initial systemic therapy before chemotherapy

Of the 59 patients with ER‐positive HER2‐positive AMBC who received endocrine therapy (including F500) or anti‐HER2 therapy as initial systemic therapy before being switched to chemotherapy, 47 (80%) received this treatment as endocrine therapy alone; 4 (7%), as anti‐HER2 therapy alone; and 8 (14%), as endocrine plus anti‐HER2 therapy (Table S1).

**FIGURE 6:**
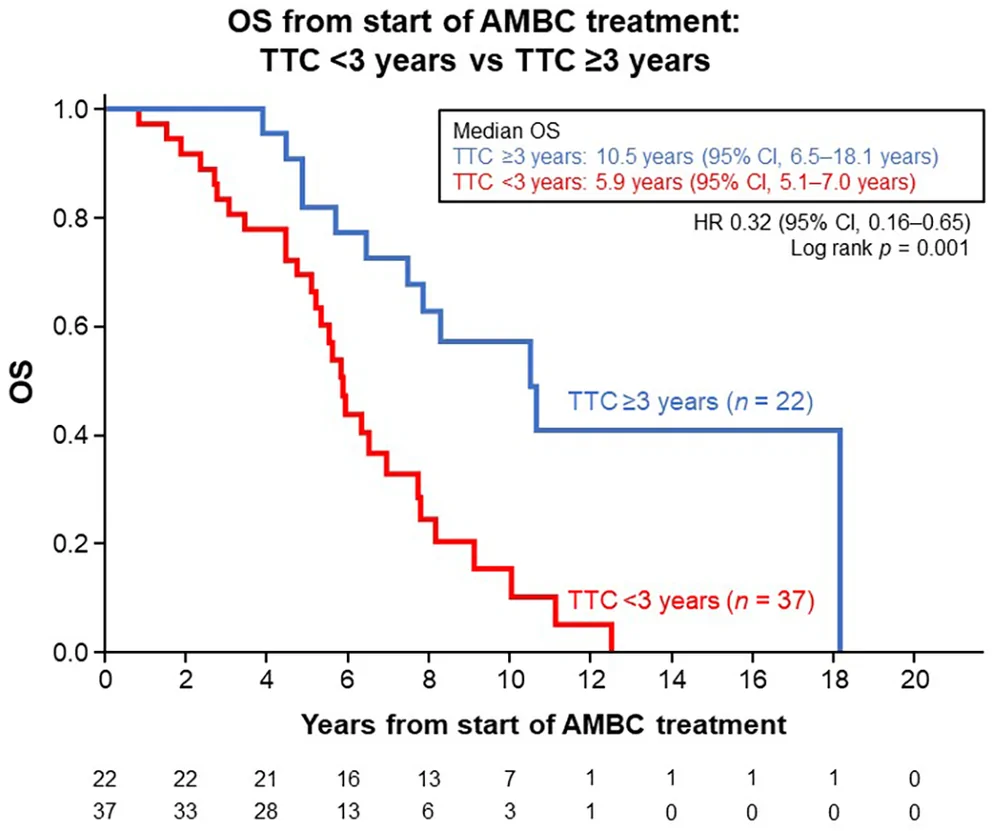
Kaplan–Meier estimates for overall survival (OS) in patients who received endocrine therapy (including fulvestrant 500 mg) or anti‐HER2 therapy as initial systemic therapy before being switched to chemotherapy (*n* = 59), from the start of advanced or metastatic breast cancer (AMBC) treatment and stratified by the cut‐off value of time to chemotherapy (TTC) 3 years. CI, confidence interval; HR, hazard ratio.

Median TTC in the patients who received endocrine therapy and anti‐HER2 therapy as initial systemic therapy before chemotherapy was 22.7 months (range, 2.0–160.8 months). Patients with TTC ≥3 years had significantly longer median OS than those with TTC <3 years: 10.5 years (95% CI, 6.5–18.1 years) vs. 5.9 years (95% CI, 5.1–7.0 years) (HR, 0.32 [95% CI, 0.16–0.65]; *p* = 0.001) (Figure 6).

No significant differences in OS were found between patients with TTF above and below the median (8.1 months), although patients with longer TTF tended to have longer OS (Figure S2a). By contrast, TTC above the median (22.7 months) was significantly associated with prolonged OS (HR, 0.37 [95% CI, 0.19–0.71]; *p* = 0.002) (Figure S2b).

#### 3.2.3 Analysis 3: factors associated with OS

In the patients who received endocrine therapy (including F500) or anti‐HER2 therapy as initial systemic therapy for AMBC before being switched to chemotherapy (*n* = 59), the results of the univariate analysis showed longer TTC and TTF to be significantly associated with prolonged OS, whereas the results of the multivariate analysis showed only TTC as a factor significantly associated with prolonged OS (HR, 0.74 [95% CI, 0.59–0.91]; *p* = 0.008) (Table 2).

**TABLE 2:** Factors associated with overall survival in patients who received endocrine therapy (including fulvestrant 500 mg) or anti‐HER2 therapy as initial systemic therapy before being switched to chemotherapy (*n* = 59): results of univariate and multivariate Cox proportional hazards regression models.

Explanatory variable	Univariate analysis			Multivariate analysis		
	HR	95% CI	*p*	HR	95% CI	*p*
Age at AMBC diagnosis (continuous quantity)	1.03	0.99–1.06	0.19	1.03	0.99–1.08	0.16
Visceral metastasis (no vs. yes)	0.58	0.28–1.23	0.15	0.49	0.22–1.07	0.07
PgR expression (positive vs. negative)	1.13	0.58–2.22	0.72	1.43	0.68–3.00	0.34
HER2 expression (weak positive vs. strong positive)	0.58	0.29–1.16	0.12	0.46	0.19–1.11	0.08
Time to chemotherapy (continuous quantity)	0.77	0.66–0.90	0.001*	0.74	0.59–0.91	0.008*
Time to treatment failure (continuous quantity)	0.65	0.44–0.90	0.02*	0.98	0.60–1.55	0.93
Endocrine plus anti‐HER2 therapy (no vs. yes)	1.07	0.56–2.04	0.83	0.81	0.33–2.01	0.65

Abbreviations: AMBC, advanced/metastatic breast cancer; CI, confidence interval; HER2, human epidermal growth factor receptor 2; HR, hazard ratio; PgR, progesterone receptor.

* *p* < 0.05.

When data from the 8 patients with de novo stage IV cancer were excluded, the results of univariate analysis of data from the remaining 51 patients showed longer TTC to be significantly associated with prolonged OS (HR, 0.80 [95% CI, 0.68–0.94]; *p* = 0.006). Factors shown by the results of multivariate analysis to be significantly associated with prolonged OS were younger age at AMBC diagnosis (HR, 1.07 [95% CI, 1.01–1.13]; *p* = 0.02) and strong HER2 expression (HR, 0.35 [95% CI, 0.15–0.81]; *p* = 0.01) (Table S2).

For reference, in the total population of ER‐positive HER2‐positive AMBC patients who received F500 with or without anti‐HER2 therapy (*n* = 94), the results of both univariate and multivariate analysis showed younger age at diagnosis of AMBC to be a factor significantly associated with prolonged OS (Table S3).

## 4 DISCUSSION

### 4.1 Interpretation of TTF results

In this subgroup analysis using data from ER‐positive HER2‐positive AMBC patients enrolled in the large‐scale, retrospective cohort Safari study, the median TTF was 4.4 months (95% CI, 3.3–5.7 months). For comparison, TTF in the overall Safari study population (*n* = 1072) and HER2‐negative subgroup (*n* = 828) was 5.4 months^3^ and 5.39 months,^5^ respectively.

Our analysis showed that TTF was significantly longer in patients treated with F500 as first‐ or second‐line therapy than in those who received it as third‐ or later‐line therapy (6.6 months vs. 3.7 months). This suggests that the necessity for the use of advanced treatment lines indicates resistance to both endocrine and anti‐HER2 therapies.

### 4.2 Comparison with other studies

Few data are available regarding the efficacy of fulvestrant in patients with ER‐positive HER2‐positive AMBC, and therefore, its role in the treatment of this population is unclear. Although differences between previous studies (e.g., in terms of patients' menopausal status, fulvestrant doses used, and assessment of outcomes) preclude their direct comparison, we here present the following data for reference.

In a pooled analysis of data from 102 postmenopausal women with ER‐positive HER2‐positive AMBC treated with fulvestrant (250 mg monthly; 5 patients received concomitant trastuzumab), the median duration of fulvestrant treatment was 8.1 months.^11^ At the start of fulvestrant treatment, patients had been treated using a median of 1.5 prior endocrine therapy and 1.8 chemotherapy regimens.^11^

In the retrospective HERMIONE 9 study, in which real‐world data from 87 patients (including 60 postmenopausal patients) with ER‐positive HER2‐positive advanced breast cancer treated with F500 plus trastuzumab were analyzed, progression‐free survival (PFS) was 12.9 months and 77% of patients had been treated using three or more previous therapies (both chemotherapy and endocrine therapy with or without anti‐HER2 therapy).^12^ Treatment with F500 plus trastuzumab resulted in favorable outcomes even when compared with the combination of nonsteroidal AIs plus trastuzumab, which is generally used in earlier treatment lines.^13^, ^14^, ^15^ These data indicate that fulvestrant tends to be used in later treatment lines (mostly third or later), and that its efficacy is maintained even when it is used following disease progression after prior endocrine and anti‐HER2 therapies and after chemotherapy.^11^, ^12^

### 4.3 Usefulness of F500 with or without anti‐HER2 therapy as initial (chemotherapy‐free) systemic therapy

The standard first‐line treatment for HER2‐positive AMBC is anti‐HER2 therapy plus chemotherapy.^6^, ^7^ However, in patients with ER‐positive disease, endocrine‐based therapy may be a useful treatment option to maintain a good quality of life (at least partly from avoidance of the adverse effects of chemotherapy) and prolong OS. This is what we often find in clinical practice: patients who respond to the initial endocrine‐based therapy are likely to have a good prognosis. The results of the present subgroup analysis are, to our knowledge, the first to show prolonged OS in patients with ER‐positive HER2‐positive AMBC who have responded to initial endocrine or anti‐HER2 therapy.

According to the Japanese Breast Cancer Society Clinical Practice Guidelines for systemic treatment of breast cancer, endocrine therapy combined with anti‐HER2 therapy may be considered for patients with ER‐positive HER2‐positive metastatic breast cancer and for whom chemotherapy is contraindicated.^7^

The efficacy of sequential use of endocrine therapy with or without anti‐HER2 therapy for the treatment of AMBC remains uncertain. Because nonsteroidal AIs are widely used in the adjuvant setting for postmenopausal patients with ER‐positive disease, F500 may be an option for first‐line endocrine therapy in cases of recurrence or progression despite adjuvant AI therapy.^16^

In the Safari study, 59 (63%) of 94 ER‐positive HER2‐positive patients received endocrine therapy (including F500) or anti‐HER2 therapy as initial systemic therapy for AMBC before being switched to chemotherapy. In this population, the median TTF and median TTC were 8.1 months and 22.7 months, respectively. Longer TTC (≥3 years) was found to be significantly associated with prolonged OS, suggesting that this cut‐off value may be a useful indicator of clinical outcomes. Such cut‐off values are useful when physicians discuss treatment options with patients. Because of the current scarcity of evidence in this population, these findings from real‐world data may be useful and contribute to the literature on the potential use of initially chemotherapy‐free regimens.

In the present study, some patients did not receive anti‐HER2 therapy as a first‐line treatment, presumably because of concomitant or underlying disease or conditions. However, detailed reasons were not collected in the Safari study. It should be noted that when the Safari study was conducted, physicians generally followed the concept of the Hortobagyi algorithm^17^; accordingly, for patients who were susceptible to endocrine therapy and without a life‐threatening condition, the first‐line treatment was endocrine therapy alone. However, around that time, the results of the TAnDEM^13^ and EGF3008^14^ studies were published, and there was controversy regarding whether anti‐HER2 therapy should be used as first‐line treatment in combination with endocrine therapy or in later treatment lines in combination with chemotherapy. In the more recent PERTAIN study, however, the use of anti‐HER2 therapy in combination with endocrine therapy has been shown to result in significantly longer PFS.^15^ Our finding of an association between longer TTC and longer OS may be interpreted as evidence in favor of the current recommendation that anti‐HER2 therapy should be initiated early, in combination with endocrine therapy.

### 4.4 Factors associated with OS

Multivariate analysis identified longer TTC as a factor predicting prolonged OS. Thus, our results confirm that longer TTC is a good predictor of survival. Additionally, our results showed younger age to be significantly associated with prolonged OS. This finding is consistent with those reported for the overall Safari study population, in which age < 60 years correlated positively with prolonged OS (median 7.0 years).^4^ Other similarly correlated factors reported previously were a longer period between diagnosis and fulvestrant use (≥3 years), no prior palliative chemotherapy before fulvestrant use, progesterone receptor‐negative status, and lower histological or nuclear grade.^4^

### 4.5 Clinical implications

At the time of the Safari study, cyclin‐dependent kinase 4 and 6 (CDK4/6) inhibitors were unavailable. However, one finding of the present subgroup analysis, namely that patients with HER2‐positive breast cancer that is sensitive to endocrine therapy (as shown by their longer TTF) generally have longer OS, suggests that the addition of a CDK 4/6 inhibitor to endocrine therapy may benefit postmenopausal patients with ER‐positive HER2‐positive AMBC and who are not candidates for chemotherapy; the combination of a CDK 4/6 inhibitor plus endocrine therapy would avoid the risk of adverse effects associated with chemotherapy. Other nonchemotherapeutic options for treatment of ER‐positive HER2‐positive advanced breast cancer include the combination of endocrine therapy plus anti‐HER2 therapy (single‐agent or dual blockade).^10^, ^18^

In patients with hormone receptor‐positive HER2‐negative advanced breast cancer, addition of CDK4/6 inhibitors (e.g., palbociclib, abemaciclib, or ribociclib) to endocrine therapy has been shown to improve PFS, enabling a prolonged endocrine therapy‐based period and resulting in favorable OS (as shown by the results of the MONARCH, MONALEESA, and PALOMA trials).^19^, ^20^, ^21^, ^22^ In such patients, including those enrolled in the Safari study,^23^ longer time from diagnosis to chemotherapy has been found to be associated with longer OS. However, in patients with HER2‐positive breast cancer, for which the combination of chemotherapy and anti‐HER2 therapy is currently the standard first‐line treatment,^24^, ^25^ insufficient data are available to enable a consensus to be reached regarding the role of endocrine therapy in the subset of patients with ER‐positive tumors. Furthermore, because the outcomes of HER2‐positive primary breast cancer have substantially improved with the use of dual‐HER2 blockade and trastuzumab emtansine regimens (as shown by the results of the Aphinity and Katherine trials),^26^, ^27^ and the number of recurrent cases is decreasing, it is difficult to carry out prospective clinical trials to investigate the role of endocrine therapy in cases of ER‐positive HER2‐positive AMBC.

Against this background, we believe that the findings of our subgroup analysis, in which data from the retrospective cohort Safari study were used to investigate the effectiveness of F500 in a real‐world clinical setting, may provide useful information for clinicians treating patients with ER‐positive HER2‐positive AMBC. We found that patients in a postmenopausal hormonal state and with ER‐positive HER2‐positive AMBC, who received F500 with or without anti‐HER2 therapy, TTF was longer in the patients treated with F500 as first‐ or second‐line therapy than in those who received later‐line F500 therapy. We also found that in the patients who received endocrine therapy or anti‐HER2 therapy as initial systemic therapy before chemotherapy, those with TTC ≥3 years had longer median OS than those with TTC <3 years. These findings suggest that if their disease condition permits, endocrine therapy plus anti‐HER2 therapy may be considered a useful treatment option, providing better quality of life, for patients with ER‐positive HER‐positive AMBC.

### 4.6 Research implications

The present study provides original data on the use of targeted agents early in the care of Japanese patients with ER‐positive HER2‐positive AMBC, in terms of the clinical outcomes achieved using this treatment approach. The improvements in both TTF and OS found in the patients who received targeted therapy (endocrine therapy or anti‐HER2 therapy) before chemotherapy support further research on the efficacy and safety of upfront targeted agents (CDK4/6 inhibitors, endocrine therapy, and/or anti‐HER2 therapy), or the earlier integration of such agents, used before chemotherapy.

### 4.7 Limitations

As with the original Safari study, there are several limitations because of the retrospective nature of the study, including the absence of a comparative treatment group and use of TTF instead of PFS. As explained previously,^3^, ^5^ TTF was used as an endpoint in the Safari study because it was difficult to retrospectively obtain PFS data as determined by the RECIST criteria, and F500 was expected to be used as third‐ or later‐line treatment in many cases. In the Safari study,^3^ definitions of treatment failure included change of treatment due to adverse effects; in some cases, therefore, the treatment could have been effective but not tolerated.

The present subgroup analysis is limited by the small sample size, which is attributable to the small number of ER‐positive HER2‐positive AMBC patients included in the Safari study. Some of the patients had undergone surgery for primary tumors before anti‐HER2 therapy became available and, therefore, could not receive anti‐HER2 therapy during the perioperative period.

Furthermore, because in the present study enrollment of patients started shortly after the approval of F500, physicians may have selected F500 for patients who had developed resistance to an AI‐based regimen, even when endocrine therapy may have been an option. Whether AI or F500 is preferable as a first‐line treatment is an important clinical question that needs further investigation.

Performance status (PS) could have been a confounding factor in the present study because it potentially affects the prognosis and treatment outcome of patients with AMBC. However, we were unable to adjust for the influence of PS because our retrospective analysis relied on real‐world data with incomplete PS information; in daily clinical practice, PS data are rarely documented in the medical record.

## 5 CONCLUSIONS

In this subgroup analysis of the Safari study of patients in a postmenopausal state and with ER‐positive HER2‐positive AMBC, TTF was longer in patients who received F500 as first‐ or second‐line therapy. In the patients who received endocrine therapy (including F500) or anti‐HER2 therapy as initial systemic therapy before being switched to chemotherapy, the prolonged OS was observed in patients with TTC ≥3 years suggesting that this may be a helpful cut‐off value for indicating clinical outcomes.

## AUTHOR CONTRIBUTIONS

**Misato Masuyama:** Conceptualization (lead); data curation (equal); formal analysis (equal); investigation (equal); methodology (equal); writing – original draft (lead); writing – review and editing (lead). **Norikazu Masuda:** Conceptualization (equal); data curation (equal); funding acquisition (equal); investigation (equal); methodology (equal); project administration (equal); resources (equal); supervision (lead); writing – original draft (equal); writing – review and editing (equal). **Hidetoshi Kawaguchi:** Conceptualization (equal); data curation (equal); investigation (equal); methodology (equal); project administration (equal); writing – original draft (equal); writing – review and editing (equal). **Yutaka Yamamoto:** Conceptualization (equal); data curation (equal); investigation (equal); methodology (equal); writing – review and editing (equal). **Shigehira Saji:** Data curation (equal); investigation (equal); writing – review and editing (equal). **Takahiro Nakayama:** Conceptualization (equal); data curation (equal); investigation (equal); methodology (equal); writing – review and editing (equal). **Kenjiro Aogi:** Data curation (equal); investigation (equal); writing – review and editing (equal). **Keisei Anan:** Data curation (equal); investigation (equal); writing – review and editing (equal). **Shoichiro Ohtani:** Data curation (equal); investigation (equal); writing – review and editing (equal). **Nobuaki Sato:** Data curation (equal); investigation (equal); writing – review and editing (equal). **Toshimi Takano:** Data curation (equal); investigation (equal); writing – review and editing (equal). **Eriko Tokunaga:** Data curation (equal); investigation (equal); writing – review and editing (equal). **Seigo Nakamura:** Data curation (equal); investigation (equal); writing – review and editing (equal). **Yoshie Hasegawa:** Data curation (equal); investigation (equal); writing – review and editing (equal). **Masaya Hattori:** Data curation (equal); investigation (equal); writing – review and editing (equal). **Tomomi Fujisawa:** Data curation (equal); investigation (equal); writing – review and editing (equal). **Satoshi Morita:** Conceptualization (equal); data curation (equal); formal analysis (equal); methodology (equal); writing – review and editing (equal). **Miki Yamaguchi:** Data curation (equal); investigation (equal); writing – review and editing (equal). **Toshinari Yamashita:** Data curation (equal); investigation (equal); writing – review and editing (equal). **Daisuke Yotsumoto:** Data curation (equal); investigation (equal); writing – review and editing (equal). **Masakazu Toi:** Conceptualization (equal); data curation (equal); investigation (equal); methodology (equal); writing – review and editing (equal). **Shinji Ohno:** Data curation (equal); investigation (equal); writing – review and editing (equal).

## FUNDING INFORMATION

Funding for this study was provided by the JBCRG and AstraZeneca.

## CONFLICT OF INTEREST STATEMENT

Masuda N received grants to his institution from Chugai Pharmaceutical, Eli Lilly, AstraZeneca, Pfizer, Daiichi Sankyo, MSD, Eisai, Novartis Pharma, Sanofi, Kyowa Kirin, Nippon Kayaku; received personal fees including honoraria from Chugai Pharmaceutical, Pfizer, AstraZeneca, Eli Lilly, and Eisai; is a member of the board of directors (unpaid) for Japan Breast Cancer Research Group (JBCRG) and Japanese Breast Cancer Society (JBCS). Kawaguchi H received support including funding from AstraZeneca and JBCRG; received consulting fees from AstraZeneca, Chugai Pharmaceutical, Eisai, and Pfizer; received personal fees (honoraria for lectures) from Pfizer, Chugai Pharmaceutical, AstraZeneca, Eisai, Kyowa Kirin, Novartis Pharma, Maruho, Eli Lilly, Taiho Pharmaceutical, Daiichi Sankyo and Nippon Kayaku. Yamamoto Y received grants to his institution from Chugai Pharmaceutical, Kyowa Kirin, Eisai, Daiichi Sankyo, Nippon Kayaku, Taiho Pharmaceutical, Takeda Pharmaceutical, Eli Lilly, Pfizer, and Novartis Pharma; received personal fees (honoraria for lectures) from AstraZeneca, Chugai Pharmaceutical, Kyowa Kirin, Novartis Pharma, Eli Lilly, Pfizer, Daiichi Sankyo, Nippon Kayaku, Taiho Pharmaceutical, Eisai and Takeda Pharmaceutical; is a member of the advisory board for AstraZeneca, Chugai Pharmaceutical, Novartis Pharma, Eli Lilly, Pfizer, Daiichi Sankyo and a member of the board of directors for JBCRG and JBCS. Saji S received grants to his institution from Taiho Pharmaceutical, Eisai, Chugai Pharmaceutical, Takeda Pharmaceutical, MSD, AstraZeneca, Daiichi Sankyo, and Eli Lilly; received personal fees including honoraria from Chugai Pharmaceutical, Kyowa Kirin, MSD, Novartis Pharma, Eisai, Takeda Pharmaceutical, Daiichi Sankyo, Eli Lilly, AstraZeneca, Pfizer, Taiho Pharmaceutical, Ono Pharmaceutical, and Nippon Kayaku; is a member of the data safety monitoring board / advisory board for Chugai Pharmaceutical, AstraZeneca, Eli Lilly, Pfizer, Kyowa Kirin, and Daiichi Sankyo; is an executive board member for JBCRG, JBCS, Japanese Society of Medical Oncology (JSMO) and Breast International Group (BIG). Nakayama T received personal fees (honoraria for lectures) from Chugai Pharmaceutical, Eli Lilly, Novartis Pharma, AstraZeneca, Pfizer, Taiho Pharmaceutical, Daiichi Sankyo, and Eisai. Aogi K received grants to his institution from Chugai Pharmaceutical, Eisai, and Takeda Pharmaceutical; received personal fees including honoraria from AstraZeneca, Taiho Pharmaceutical, Novartis Pharma, Chugai Pharmaceutical, Daiichi Sankyo, Pfizer, and Eli Lilly. Anan K received personal fees including honoraria from Pfizer, Chugai Pharmaceutical, AstraZeneca, Eisai, and Eli Lilly. Ohtani S received personal fees including honoraria from Pfizer, Eli Lilly, Chugai Pharmaceutical, and Daiichi Sankyo. Sato N received personal fees (honoraria for lectures) from Chugai Pharmaceutical, Kyowa Kirin, Taiho Pharmaceutical, Eli Lilly, Nippon Kayaku, Daiichi Sankyo, and Celltrion Healthcare Japan. Takano T received grants to his institution from Chugai Pharmaceutical, Daiichi Sankyo, Ono Pharmaceutical, MSD, and Eisai; received personal fees (honoraria for lectures) from Chugai Pharmaceutical, Daiichi Sankyo, Eisai, Eli Lilly, and Celltrion Healthcare Japan. Tokunaga E received personal fees including honoraria from Eli Lilly, AstraZeneca, and Daiichi Sankyo. Nakamura S received grants to his institution from Chugai Pharmaceutical, Daiichi Sankyo, Eisai, Konica Minolta, and Taiho Pharmaceutical; received personal fees including honoraria from AstraZeneca, Chugai Pharmaceutical, and Eisai. Hattori M received personal fees including honoraria from Eli Lilly and Daiichi Sankyo. Morita S received grants to his institution from Eisai; received personal fees including honoraria from AstraZeneca, Bristol Myers Squibb, Chugai Pharmaceutical, Eisai, Eli Lilly, MSD, Pfizer, Taiho Pharmaceutical, and Novartis Pharma. Yamaguchi M received personal fees (honoraria for lectures) from Pfizer, Chugai Pharmaceutical, Novartis Pharma, Eli Lilly, Daiichi Sankyo, and Taiho Pharmaceutical. Yamashita T received grants to his institution from Nippon Kayaku, Chugai Pharmaceutical, Taiho Pharmaceutical, and Kyowa Kirin; received personal fees (honoraria for lectures) from Nippon Kayaku, Chugai Pharmaceutical, Daiichi Sankyo, Eisai, Eli Lilly, Novartis Pharma, Taiho Pharmaceutical, AstraZeneca, Pfizer, and Kyowa Kirin. Toi M received grants to his institution from Chugai Pharmaceutical, Takeda Pharmaceutical, Pfizer, Kyowa Kirin, Taiho Pharmaceutical, JBCRG, Eisai, Daiichi Sankyo, AstraZeneca, Astellas, Shimadzu, Yakult, Nippon Kayaku, AFI technology, Luxonus, and Shionogi; received personal fees (honoraria for lecture/lecture chair) from Chugai Pharmaceutical, Takeda Pharmaceutical, Pfizer, Kyowa Kirin, Taiho Pharmaceutical, Eisai, Daiichi Sankyo, AstraZeneca, Eli Lilly, MSD, Exact Science, Novartis Pharma, Konica Minolta, Shimadzu, Yakult, and Nippon Kayaku; is a member of the advisory board for Kyowa Kirin, Daiichi Sankyo, Eli Lilly, Konica Minolta, Bristol Myers Squibb, and Athenex Oncology; is a member of the board of directors (unpaid) for JBCRG, Kyoto Breast Cancer Research Network (KBCRN) and Organization for Oncology and Translational Research (OOTR). Ohno S received a grant to his institution from Eisai; received personal fees including honoraria from Chugai Pharmaceutical, AstraZeneca, Eisai, Pfizer, and Eli Lilly. Masuyama M, Hasegawa Y, Fujisawa T, and Yotsumoto D have no conflict of interest to declare regarding the present study.

## ETHICAL APPROVAL STATEMENT

The study was conducted in compliance with the Declaration of Helsinki, “Guidelines for Clinical Evaluation Methods of Anti‐Cancer Drugs,” and “Ethical Guidelines for Epidemiology Research (revised on December 1, 2008).” The protocol was approved by the Institutional Ethics Committee at each study site. The requirement for informed consent was waived because of the retrospective, anonymous nature of the data collection.

## CLINICAL TRIAL REGISTRATION NUMBER

The study is registered with the UMIN Clinical Trials Registry (http://www.umin.ac.jp/ctr/index‐j.htm), under the unique ID UMIN000015168.

## Supporting information


Figure S1.

Figure S2.

Table S1–S3.

## Data Availability

The data are available on request due to privacy/ethical restrictions.
