# Supplementary figures and images for: Fulvestrant with or without anti‐HER2 therapy in patients in a postmenopausal hormonal state and with ER‐positive HER2‐positive advanced or metastatic breast cancer: A subgroup analysis of data from the Safari study (JBCRG‐C06)
Source: Cancer Med. 2023 Aug 1;12(17):17718–30. doi: 10.1002/cam4.6390 (PMC10523974; doi:10.1002/cam4.6390)

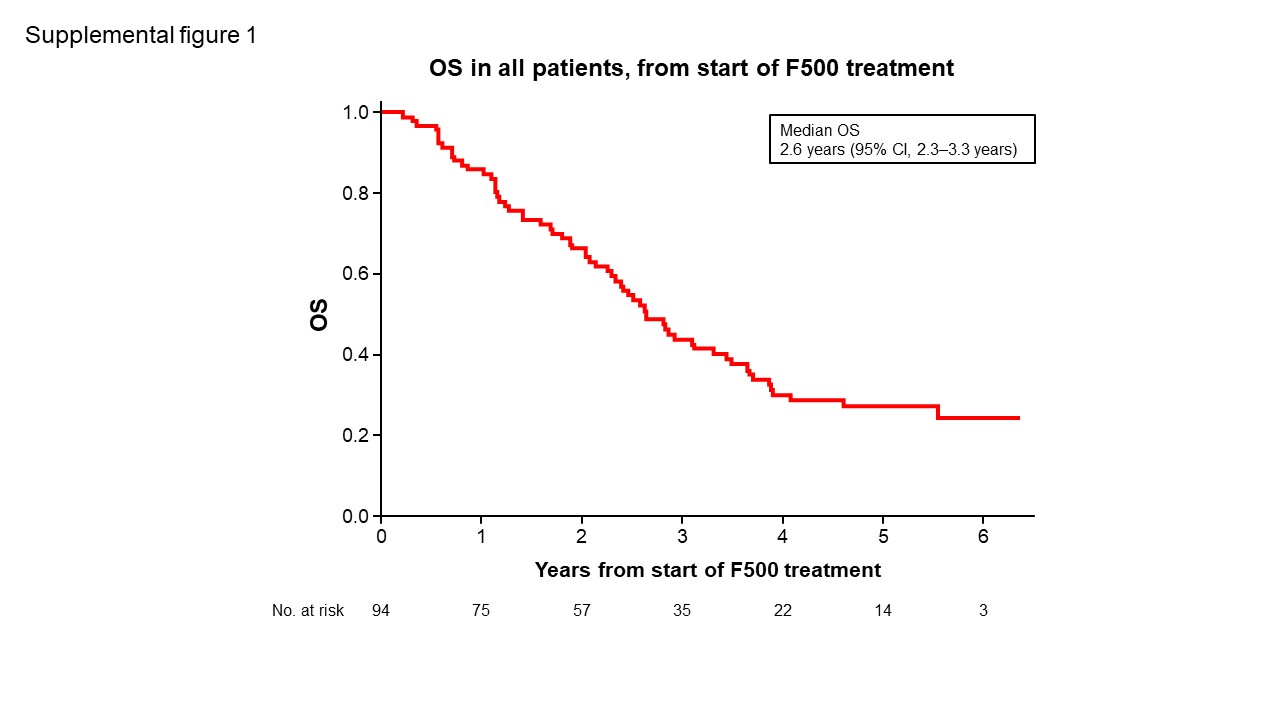

Supplement: Supplementary file 1 — Figure S1. [file CAM4-12-17718-s001.zip › Supp Fig.1.JPG]

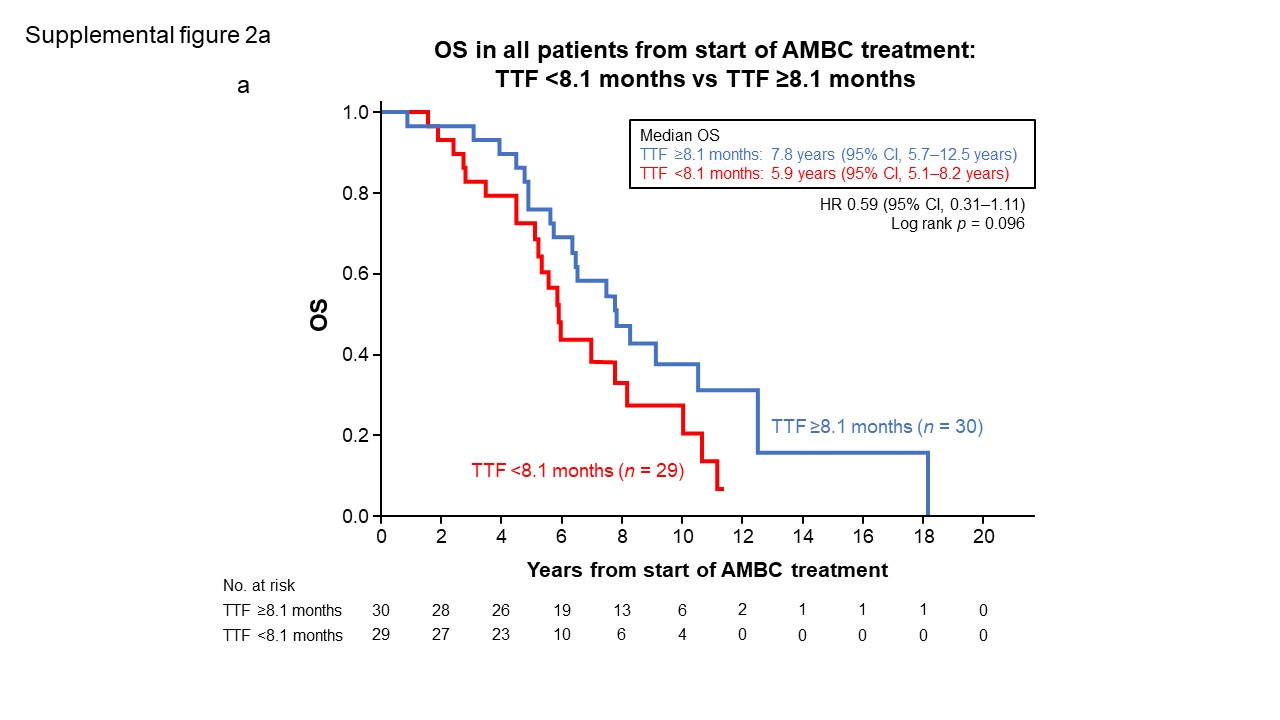

Supplement: Supplementary file 2 — Figure S2. [file CAM4-12-17718-s003.zip › Supp fig.2a.JPG]

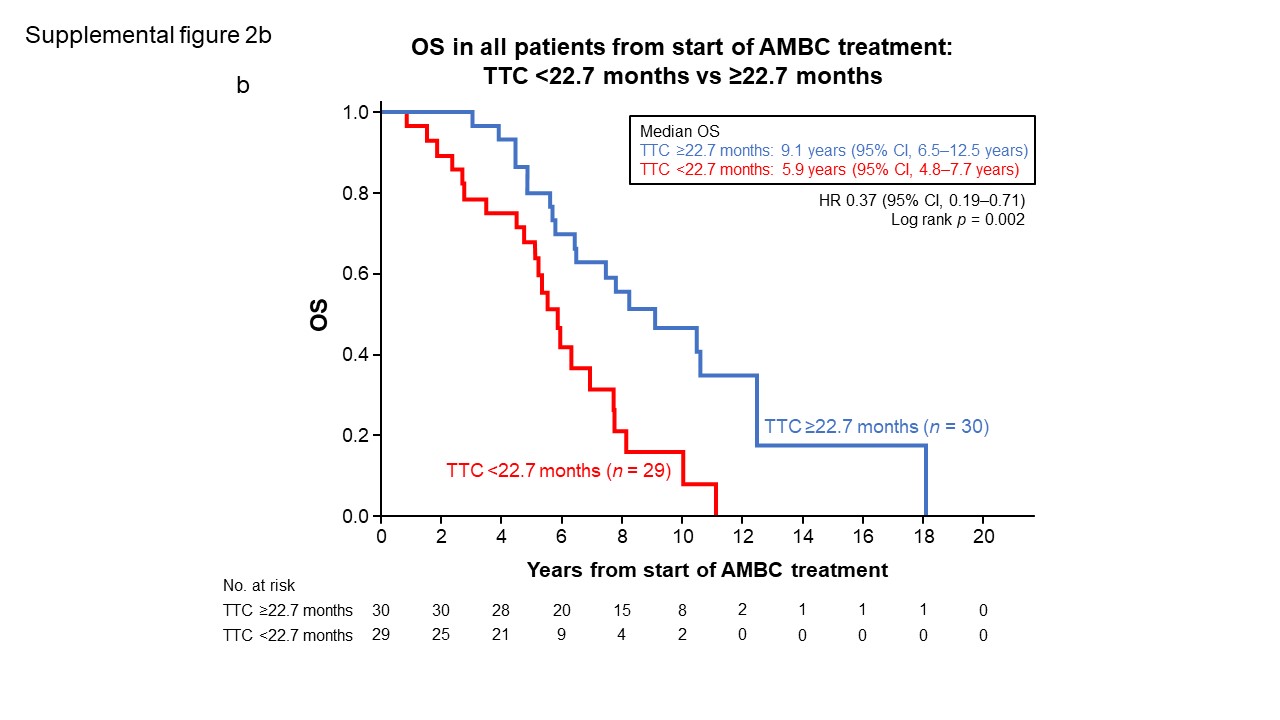

Supplement: Supplementary file 2 — Figure S2. [file CAM4-12-17718-s003.zip › Supp fig.2b.JPG]
