# Supplementary material for: Fulvestrant with or without anti‐HER2 therapy in patients in a postmenopausal hormonal state and with ER‐positive HER2‐positive advanced or metastatic breast cancer: A subgroup analysis of data from the Safari study (JBCRG‐C06)
Source: Cancer Med. 2023 Aug 1;12(17):17718–30. doi: 10.1002/cam4.6390 (PMC10523974; doi:10.1002/cam4.6390)
Supplement: Supplementary file 3 — Table S1–S3. [file CAM4-12-17718-s002.docx]

**Supplemental table 1.** Characteristics of patients who received endocrine or anti-HER2 therapy as initial systemic therapy†

|  | All (*n* = 59) | Patients with recurrent metastatic cancer (*n* = 51) | Patients with *de novo* metastatic or locally advanced cancer (*n* = 8) |
| --- | --- | --- | --- |
| Median age at AMBC diagnosis, years (range) | 58 (33–84) | 57 (33–75) | 64 (44–84) |
| Stage (first examination) |  |  |  |
| 0 | 0 | 0 | 0 |
| Ⅰ | 6 (10) | 6 (12) | NA |
| Ⅱ | 33 (56) | 33 (65) | NA |
| Ⅲ | 7 (12) | 7 (14) | NA |
| Ⅳ | 8 (14) | 0 | 8 (100) |
| NA | 5 (9) | 5 (10) | NA |
| Visceral metastasis at diagnosis |  |  |  |
| No | 41 (70) | 37 (73) | 4 (50) |
| Yes | 18 (31) | 14 (28) | 4 (50) |
| Hormonal receptor status |  |  |  |
| ER(+) PgR(–) | 15 (25) | 14 (28) | 1 (13) |
| ER(+) PgR(+) | 44 (75) | 37 (73) | 7 (88) |
| HER2 |  |  |  |
| 3+ | 23 (39) | 21 (41) | 2 (25) |
| 2+ or 1+ (positive by FISH) | 26 (44) | 25 (49) | 1 (13) |
| Positive (score unknown) | 10 (17) | 5 (10) | 5 (63) |
| Median disease-free interval, years (range) | NA | 4.6 (1–13) | NA |
| Initial systemic treatment after AMBC diagnosis |  |  |  |
| Endocrine therapy | 47 (80) | 41 (80) | 6 (75) |
| Anti-HER2 therapy | 4 (7) | 4 (8) | 0 |
| Endocrine therapy plus anti-HER2 therapy | 8 (14) | 6 (12) | 2 (25) |
| Median TTC, months (range) | 22.7 (2.0–160.8) | 20.6 (2.0–160.8) | 46.0 (7.8–134.8) |
| Median TTF for initial endocrine or endocrine plus anti-HER2 therapy, months (range) | 8.1 (1.7–91.2) | 7.7 (1.7–91.2) | 15.5 (2.9–37.4) |

AMBC, advanced/metastatic breast cancer; ER, estrogen receptor; FISH, fluorescence in-situ hybridization; HER2, human epidermal growth factor receptor 2; NA, not assessed; PgR, progesterone receptor; TTC, time to chemotherapy; TTF, time to treatment failure

† Values presented as *n* (%) or *n*, unless otherwise indicated.

**Supplemental table 2.** Factors associated with overall survival in patients who received endocrine therapy (including fulvestrant 500 mg) or anti-HER2 therapy as initial systemic therapy before chemotherapy (*n* = 51, after exclusion of data from patients with *de novo* stage IV cancer): results of univariate and multivariate Cox proportional hazards regression models

| Explanatory variable | Univariate analysis | | | Multivariate analysis | | |
| --- | --- | --- | --- | --- | --- | --- |
|  | HR | 95% CI | *p* | HR | 95% CI | *p* |
| Age at AMBC diagnosis (continuous quantity) | 1.05 | 1.00–1.10 | 0.057 | 1.07 | 1.01–1.13 | 0.02* |
| Visceral metastasis (no vs yes) | 0.57 | 0.25–1.31 | 0.19 | 0.45 | 0.18–1.10 | 0.08 |
| PgR expression (positive vs negative) | 1.25 | 0.62–2.52 | 0.54 | 2.35 | 0.95–5.81 | 0.07 |
| HER2 expression (weak positive vs strong positive) | 0.55 | 0.27–1.12 | 0.10 | 0.35 | 0.15–0.81 | 0.01* |
| Time to chemotherapy (continuous quantity) | 0.80 | 0.68–0.94 | 0.006* | 0.86 | 0.71–1.06 | 0.15 |
| Disease-free interval (continuous quantity) | 0.92 | 0.83–1.02 | 0.11 | 0.90 | 0.77–1.05 | 0.16 |

AMBC, advanced/metastatic breast cancer; CI, confidence interval; HER2, human epidermal growth factor receptor 2; HR, hazard ratio; PgR, progesterone receptor.

* *p* < 0.05.

**Supplemental table 3.** Factors associated with overall survival in all patients (*n* = 94): results of univariate and multivariate Cox proportional hazards regression models

| Explanatory variable | Univariate analysis | | | Multivariate analysis | | |
| --- | --- | --- | --- | --- | --- | --- |
|  | HR | 95% CI | *p* | HR | 95% CI | *p* |
| Age at AMBC diagnosis (continuous quantity) | 1.03 | 1.01–1.05 | 0.02* | 1.04 | 1.01–1.06 | 0.003* |
| Visceral metastasis  (no vs yes) | 0.75 | 0.44–1.27 | 0.29 | 0.63 | 0.37–1.09 | 0.098 |
| PgR expression (positive vs negative) | 1.17 | 0.67–2.02 | 0.59 | 1.47 | 0.83–2.61 | 0.19 |
| HER2 expression  (weak positive vs strong positive) | 1.08 | 0.62–1.90 | 0.78 | 1.05 | 0.58–1.87 | 0.88 |

CI, confidence interval; HR, hazard ratio; PgR, progesterone receptor.

* *p* < 0.05.
